# Multi-omics analysis reveals gut microbial and metabolic signatures in metabolic dysfunction-associated steatotic liver disease

**DOI:** 10.3389/fmicb.2025.1666110

**Published:** 2025-11-06

**Authors:** Muyesaier Maimaitiyiming, Sulitanguli Maihemuti, Tangnuer Aierken, Gulizhati Abulimiti, Tuersinayi Aibaidula, Yaru Guan, Abudoukeyoumu Simayi, Maiwulan Aimaiti, Xingfu Wang, Abula Abuduaini, Yierpan Aishan

**Affiliations:** 1 Department of Gastroenterology, The First People’s Hospital of Kashgar Prefecture, Kashgar, Xinjiang, China; 2 Department of Imaging Medicine, The Second People’s Hospital of Kashgar Prefecture, Kashgar, Xinjiang, China; 3 Department of Gastroenterology, Jiashi County People’s Hospital of Kashgar Prefecture, Kashgar, Xinjiang, China

**Keywords:** MASLD, 16S rDNA sequence, untargeted metabolomics, gut microbiota, glycerophospholipid

## Abstract

**Introduction:**

The high incidence rate of metabolic dysfunction-associated steatotic liver disease (MASLD) has been a big burden on public health globally.

**Methods:**

To explore microbial and metabolic characteristics of MASLD, we performed 16S rDNA sequencing and untargeted metabolomics on 138 stool samples from MASLD patients. Through the construction of multi-omics featuremaps, we identified relevant changes in microbial and metabolic signatures and evaluated potential clinical value in MASLD.

**Results:**

The result showed that the high-fat, high-protein dietary pattern in MASLD patients is one of the reasons for the upregulation of *Parabacteroides merdae* abundance. And it can increase the branched-chain amino acid catabolic capacity in MASLD patients, thereby improving metabolic syndrome and increasing the abundance of beneficial bacteria to improve the intestinal microbiota balance. Then, the downregulation of *Lachnospiraceae bacterium* in MASLD patients may lead to intestinal inflammatory responses. Moreover, its increasing abundance might result in heightened appetite in MASLD patients, which leads to insulin resistance and liver damage. And the increasing in glycerophospholipid (GP) metabolites in the gut of MASLD patients is highly correlated with metabolic disorders and disease progressionassociated with hepatic fat accumulation and inflammatory responses (AUC > 0.9). Therefore, the levels of GP metabolites in the stool of MASLD patients serve as a reliable diagnostic biomarker for fatty liver and represent a potential target for the diagnosis and treatment of MASLD.

**Discussion:**

After analysis of gut microbiota and metabolites, we found that *Lactobacillus johnsonii* down-regulated in MASLD drives 2,6-Dichlorohydroquinone accumulation, provoking toxic buildup and accelerating disease progression.

## 1 Introduction

Globally, the prevalence of metabolic dysfunction-associated steatotic liver disease (MASLD) is 29.38% and is on the rise year by year (Powell et al., 2021; Liu et al., 2022). Moreover, the incidence and prevalence of MASLD are higher in males than in females, with a global prevalence of 40% in males compared to 26% in females (Sahu et al., 2023; Younossi et al., 2023). Additionally, there are significant regional differences in the prevalence of MASLD. For example, the highest prevalence is found in South America at 75.64%, while in Asia, the prevalence is approximately 30%, which still represents a substantial healthy burden (Liu et al., 2022; Sahu et al., 2023). Furthermore, MASLD exhibits specific prevalence rates in particular populations. In patients with type 2 diabetes, the prevalence of MASLD is as high as 57.85%, and in severely obese patients undergoing bariatric surgery, the prevalence reaches 82.16% (Godoy-Matos et al., 2020; Liu et al., 2022).

MASLDremains a significant burden on public health, as it not only damageshealthy liver function but is also closely associated with a variety of severe health issues (Huby and Gautier, 2022). Firstly, it can lead to the progression of liver diseases. Approximately 46.49% of MASLD patients will progress to non-alcoholic steatohepatitis (NASH), and about 46.72% of NASH patients will further develop liver fibrosis or cirrhosis (Huang et al., 2021; Powell et al., 2021). Moreover, the incidence of hepatocellular carcinoma (HCC) in MASLD patients is 1.46‰ (Anstee et al., 2019; Huang et al., 2021). In addition to these liver-related complications, MASLD is strongly linked to the occurrence of cardiovascular diseases. Cardiovascular diseases are one of the leading causes of death in MASLD patients, accounting for 40% of deaths in this patient population. Previous researches have shown that MASLD may independently increase the risk of cardiovascular diseases (Byrne and Targher, 2015; Zhang et al., 2024). Furthermore, MASLD is closely related to a number of metabolic disorders, including type 2 diabetes, obesity, hyperlipidemia, and metabolic syndrome. It is also associated with pathological mechanisms such as insulin resistance, oxidative stress, and gut microbiota dysbiosis, which collectively promote disease progression (Liu et al., 2022). Therefore, given the high incidence and prevalence of MASLD globally, as well as its extensive and severe impact on health, early identification and intervention of MASLD are crucial for improving patient outcomes.

According to analyze the gut microbiota and metabolites characteristics of MASLD for disease diagnosis and monitoring, we aim to achieve non-invasive diagnosis and monitoring of MASLD and improve personalized health management plans (Scorletti et al., 2020; Li et al., 2022). We performed 16S rDNA sequencing to analyze the gut microbiota between MASLD patients and healthy controls. Gut microbiota sequencing can comprehensively assess the diversity and balance of the intestinal microbial community. It can detect the composition and function of the microbiota. Abnormalities such as dysbiosis and enrichment of pathogenic microorganisms can be identified. This can provide guidance for early prevention and treatment (Damhorst et al., 2021; Liu et al., 2023; Song Z. et al., 2023). It can also detect microbial alterations associated with various diseases to serve as an auxiliary diagnostic tool and monitor disease progression and therapeutic efficacy. Personalized dietary plans can be developed based on the results. This can facilitate personalized health management and monitoring (Jiang et al., 2024).

Moreover, incorporating untargeted metabolomics allows for an unbiased detection of all small-molecule metabolites in biological samples, including both known and unknown ones. This provides a comprehensive understanding of metabolic changes in biological systems (Buchard et al., 2021; Babu et al., 2022; Brinca et al., 2022; Song Q. et al., 2023; Babu et al., 2024; Wang et al., 2024). Metabolites are the foundation of an organism’s phenotype and can help more directly and effectively understand biological processes and their mechanisms. Based on the qualitative and quantitative analysis of metabolites, metabolomics can be used to explain metabolic pathways or networks, investigate the metabolic basis of macroscopic phenotypic phenomena in different biological individuals, and study the response mechanisms of metabolites to various stimuli such as different diseases, drugs, or pathogenic organisms. Untargeted metabolomics is a commonly used method in metabolomics research. The main research approach is to compare experimental and control groups, detect metabolites in samples, obtain quantitative information, and identify metabolites with statistically significant differences between groups. This can explain the relationship between the identified metabolites and biological processes or states.

We performed 16S rDNA sequencing on stool samples from 80 MASLD patients and healthy controls to analyze the composition of the gut microbiota. We also performed untargeted metabolomics on stool samples from 58 MASLD patients and healthy controls to characterize the gut microbiota metabolites in MASLD patients. This approach aims to diagnose MASLD, improve non-invasive diagnostic and monitoring protocols for MASLD, and provide new guidance for the personalized health management of MASLD.

## 2 Materials and methods

### 2.1 Ethics statement

Sample collection was approved by the Ethics Committee for Scientific Research of the First People’s Hospital of Kashgar Prefecture, and informed consent was obtained from the patient. An additional number was used to register the samples in the database with no link to patient names or personal information.

### 2.2 16S ribosomal DNA sequencing of gut microbiota

Samples included 40 MASLD patients and 40 healthy controls (Ctrl). All patients were diagnosed through ultrasound examination and excluded individuals with NASH and other metabolic syndromes. MASLD patients and healthy controls were recruited from the First People’s Hospital of Kashgar Prefecture in 2023–2024.

Bacterial DNA was extracted from human stool samples using a TIANamp Stool DNA Kit (TIANGEN, Beijing, China). The purity and concentration of the DNA were then assessed by agarose gel electrophoresis. PCR was performed using barcoded primers and a high-fidelity enzyme (Phusion® High-Fidelity PCR Master Mix with GC Buffer, Biolabs, New England). The PCR protocol and primers were in Table 1. The PCR products were detected by 2%agarose gel electrophoresis. Qualified PCR products were purified with magnetic beads and then subjected to agarose gel electrophoresis again for target band recovery. Subsequently, the library was constructed using the TruSeq® DNA PCR-Free Sample Preparation Kit. The constructed library was quantified by Qubit and qPCR. After the library passed the quality check, sequencing was carried out on the NovaSeq 6,000 platform.

**Table 1:** PCR protocol and primers.

Cycle temperature time		
1 cycle	94 °C	30 s
30 cycles	94 °C	15 s
	55 °C	15–30 s
	68 °C	1 min
1 cycle	68 °C	5 min (to finish replication on all templates)
1 cycle	4–10 °C	indefinite period (storing the sample prior to further analysis)
Primers		
16S V4	515F	GTGYCAGCMGCCGCGGTAA
	806R	GGACTACNVGGGTWTCTAAT
16SV3-V4	341F	CCTACGGGNGGCWGCAG
	785R	GACTACNNGGGTATCTAATCC
16SV4-V5	515F	GTGYCAGCMGCCGCGGTAA
	926R	CCGTCAATTCMTTTRAGT
16SV5-V7	799F	AACMGGATTAGATACCCKG
	1193R	ACGTCATCCCCACCTTCC

Sample data were split from the raw sequencing data based on the Barcode sequences and PCR primer sequences, with the Barcode and primer sequences being trimmed off. Fastp (v0.22.0) was used to filter the raw reads to obtain high-quality reads. Chimeric sequences were then removed by comparison, resulting in the final Effective Tags. Subsequently, OTU clustering (USEARCH, v7), ASV denoising (Deblur, v1.1.1), and taxonomic annotation (Mothur, v1.48 and SILVA138.1) were performed. The taxonomic annotation provided classification information at various taxonomic levels, including phylum, class, order, family, genus, and species.

Moreover, alpha diversity analysis was performed to assess sample complexity. The R software (v4.2.0) with the phyloseq (v1.40.0) and vegan (v2.6.2) packages was used for this purpose. For comparing multiple samples, the LEfSe tool (v1.1.2) was utilized. In terms of functional annotation, PICRUSt2 (v2.5.0) was employed to predict metagenomic functions of the marker genes based on the KEGG database.

The raw sequence data reported in this paper have been deposited in the Genome Sequence Archive (Genomics, Proteomics & Bioinformatics 2021) in National Genomics Data Center (Nucleic Acids Res 2022), China National Center for Bioinformation/Beijing Institute of Genomics, Chinese Academy of Sciences (GSA: CRA029645) that are publicly accessible at https://ngdc.cncb.ac.cn/gsa.

### 2.3 Untargeted metabolomics

Human stool samples included 29 MASLDs and 29 healthy controls (Ctrl). MASLDs and healthy controls were recruited from the First People’s Hospital of Kashgar Prefecturein 2023–2024.

The sample preparation and extraction process for human stool samples stored at −80 °C. The samples are thawed on ice, mixed with a methanol–water solution containing an internal standard, and then sonicated and vortexed. After being placed at −20 °C for 30 min, the samples are centrifuged twice to remove sediment. The supernatant is then transferred for LC–MS analysis.

#### 2.3.1 HPLC conditions

All samples were for two LC/MS methods. One aliquot was analyzed using positive ion conditions and was eluted from T3 column (Waters ACQUITY Premier HSS T3 Column 1.8 μm, 2.1 mm × 100 mm) using 0.1% formic acid in water as solvent A and 0.1% formic acid in acetonitrile as solvent B in the following gradient: 5 to 20% in 2 min, increased to 60% in the following 3 min, increased to 99% in 1 min and held for 1.5 min, then come back to 5% mobile phase B witnin 0.1 min, held for 2.4 min. The analytical conditions were as follows, column temperature, 40 °C; flow rate, 0.4 mL/min; injection volume, 4 μL; Another aliquot was using negative ion conditions and was the same as the elution gradient of positive mode.

#### 2.3.2 MS conditions (QE)

All the methods alternated between full scan MS and data dependent MSn scans using dynamic exclusion. MS analyses were carried out using electrospray ionization in the positive ion mode and negative ion mode using full scan analysis over m/z 75–1,000 at 35000 resolution. Additional MS settings are: ion spray voltage, 3.5KV or 3.2KV in positive or negative modes, respevtively; Sheath gas (Arb), 30; Aux gas, 5; Ion transfer tube temperature, 320 °C; Vaporizer temperature, 300 °C; Collision energy, 30,40,50 V; Signal Intensity Threshold, 1*e6 cps; Top N vs. Top speed, 10; Exclusion duration, 3 s.

The data is analyzed using various statistical methods, including PCA, hierarchical cluster analysis, and differential metabolite selection. The identified metabolites are annotated using the KEGG Compound database and mapped to the KEGG Pathway database for further analysis.

### 2.4 Glutamate concentration measurement

Collect the supernatant of fecal suspension (thorough sonication-induced lysis) in MASLD and Ctrl (*n* = 5 per group). And prepare the testing reagent. Set the appropriate parameters on the automated chemistry analyzer. And load the samples. The analyzer (Chemray 800) performs the assay automatically.

## 3 Results

### 3.1 Overall gut microbiota abundance maps

**Figure 1:**
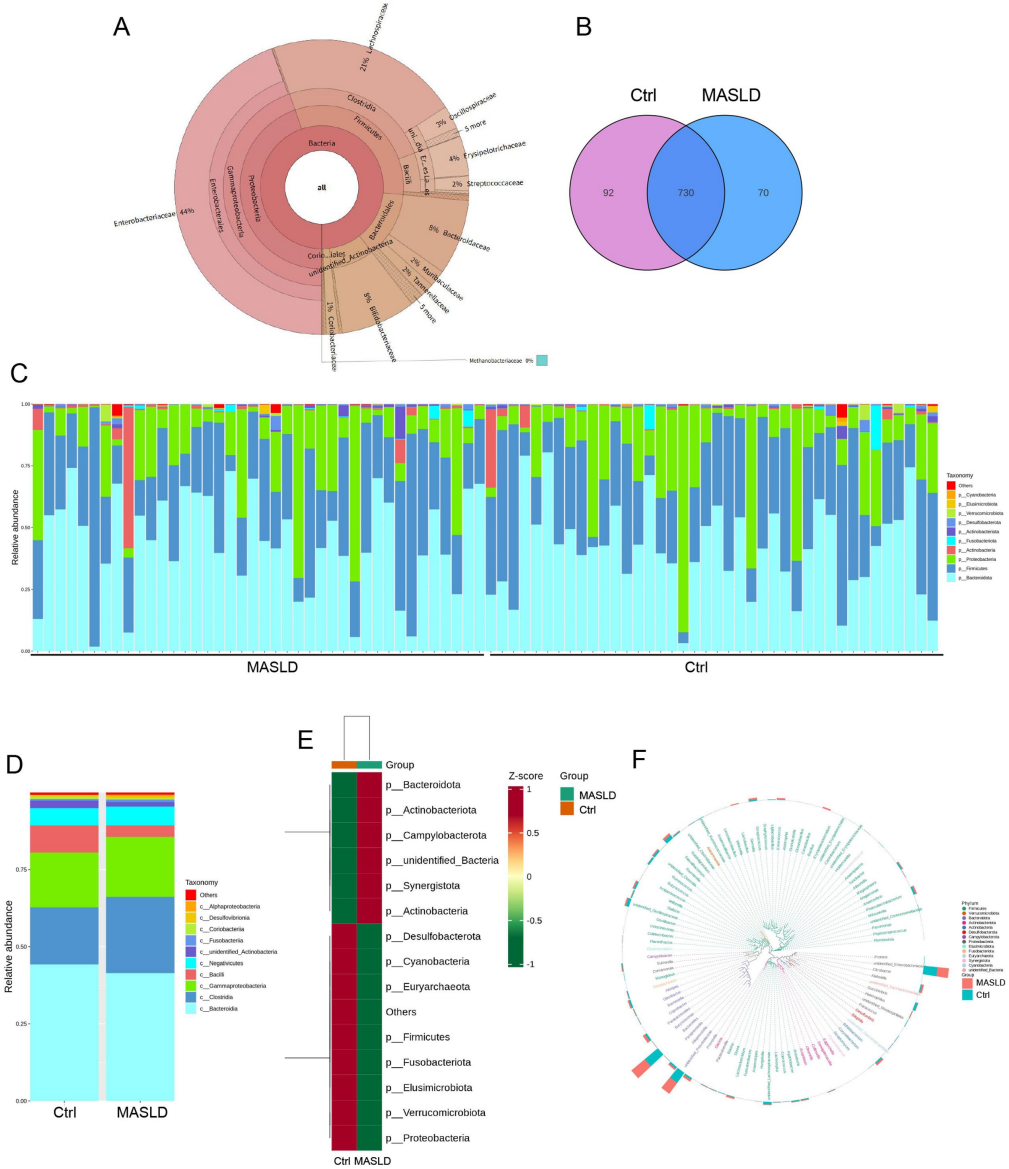
Overall gut microbiota abundance maps: **(A)** Distribution of gut microbiota species composition in 80 samples; **(B)** Venn diagram of gut microbiota in the Ctrl and MASLD groups; **(C)** Stacked bar chart of species relative abundance at the phylum level for each sample in the Ctrl and MASLD groups; **(D)** Stacked bar chart of species relative abundance at the phylum level in the Ctrl and MASLD groups; **(E)** Heatmap of species abundance at the phylum level based on ASV for different groups in the Ctrl and MASLD groups; **(F)** Phylogenetic relationships of species at the genus level based on ASV in the gut microbiota of the Ctrl and MASLD groups.

A total of 80 stool samples were collected and divided into two groups: the Metabolic Dysfunction-Associated Steatotic Liver Disease (MASLD) group and the healthy control (Ctrl) group. Table 2 showed the patient’s basic clinical data including gender, age, BMI, AST, ALT, total bilirubin and total bile acids. In these 80 samples, KRONA was used to annotate the ASV results of species. A total of 18,870 bacterial ASVs were detected. Among them, the Firmicutes accounted for 32% (5,996), the *Proteobacteria* accounted for 45% (8,434), the *Bacteroidales* accounted for 13% (2,464), and the *Coriobacteriales* accounted for 2% (304) (Figure 1A).

**Table 2:** Basic clinical data in MASLD and healthy group.

Items		MASLD	Healthy group	*p* value
Gender (%)	Male	52.5	57.5	^ns^*p* = 0.822
	Female	47.5	42.5	
Age		50.23 ± 13.13	50.10 ± 19.06	^ns^*p* = 0.973
BMI		28.63 ± 4.63	23.83 ± 4.54	^***^p < 0.001
Triglyceride (mmol/L)		2.70 ± 1.35	1.55 ± 0.88	^***^p < 0.001
Total cholesterol (mmol/L)		4.57 ± 1.11	3.90 ± 1.22	^*^p = 0.014
ALT (U/L)		40.73 ± 37.65	28.78 ± 40.69	^ns^*p* = 0.177
AST (U/L)		37.85 ± 53.48	24.48 ± 19.92	^ns^*p* = 0.145
Total bilirubin (μmol/L)		15.32 ± 27.89	10.00 ± 5.89	^ns^*p* = 0.241
Total bile acids (μmol/L)		8.85 ± 13.70	4.70 ± 4.00	^ns^*p* = 0.070

ns, *p* > 0.05; *, *p* < 0.05; **, *p* < 0.01; ***, *p* < 0.001.

There were 730 shared gut microbiota ASVs totally, with the MASLD group having 70 unique gut microbiota ASVs (Figure 1B). The top 10 most abundant species at the phylum level in each group were selected to create a stacked bar chart of species relative abundance for the 80 samples (Figure 1C). In both the MASLD and Ctrl groups, the top 5 gut microbiota were concentrated in *Bacteroidota*, *Firmicutes*, *Proteobacteria*, *Actinobacteria*, and *Fusobacteriota*. The Ctrl group had a higher proportion of *Bacteroidota* (44.25% vs. 41.46%, ^ns^*p* = 0.53), while the MASLD group had higher proportions of *Firmicutes* (34.51% vs. 32.87%, ^ns^*p* = 0.69) and *Proteobacteria* (19.91% vs. 17.89%, ^ns^*p* = 0.63) (Figure 1D). Based on ASV analysis, a heatmap of species abundance at the phylum level was created for different groups. The MASLD group was enriched in *Bacteroidota*, *Actinobacteriota*, and *Campylobacterota*, while the Ctrl group was enriched in *Proteobacteria*, *Verrucomicrobiota*, and *Elusimicrobiota* (Figure 1E). To further investigate the phylogenetic relationships of species at the genus level, the representative sequences of the top 100 genera were obtained through multiple sequence alignment. A phylogenetic tree was constructed based on the representative sequences of species at the genus level, displaying the genus-level phylogenetic tree (Figure 1F).

### 3.2 Differential microbiota maps of gut microbiota in MASLD

**Figure 2:**
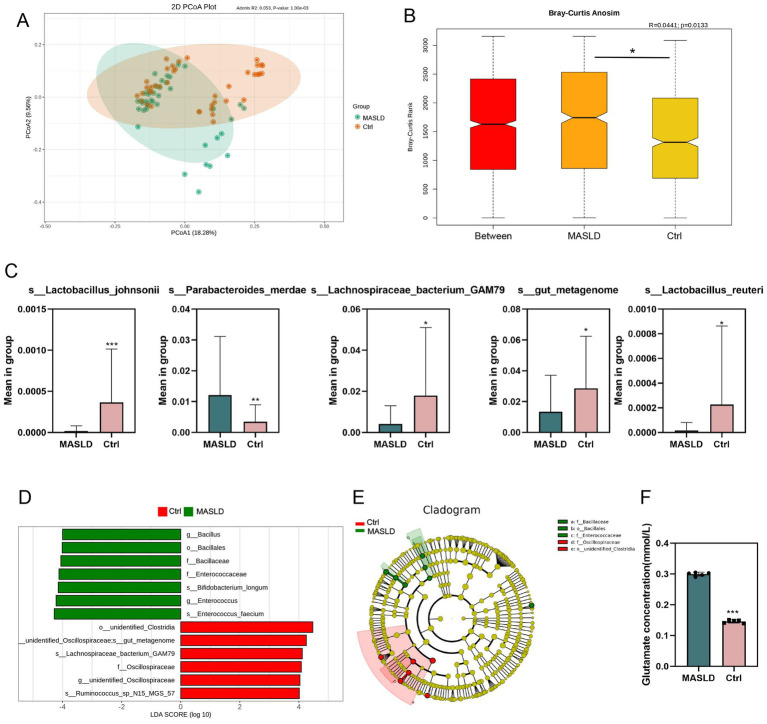
Differential microbiota maps of gut microbiota in MASLD: **(A)** PCoA plot of the Ctrl and MASLD groups; **(B)** Anosim analysis based on ASV between the Ctrl and MASLD groups; **(C)** Bar chart of species differences analysis based on ASV between the MASLD and Ctrl groups; **(D)** Bar chart of LEfSe based on ASV between the MASLD and Ctrl groups; **(E)** Phylogenetic tree based on ASV between the MASLD and Ctrl groups; **(F)** The concentration of glutamate in MASLD and Ctrl group.

Principal Co-ordinates Analysis (PCoA) was performed on the MASLD and Ctrl groups, separating the samples of the two groups (Figure 2A). Anosim analysis based on ASV showed significant differences between the MASLD and Ctrl groups (*p* = 0.013), indicating that the grouping of the MASLD and Ctrl was meaningful (Figure 2B). Differential abundance analysis identified *Parabacteroides merdae* (MASLD vs. Ctrl, 0.012 ± 0.019 vs. 0.004 ± 0.006, *p* = 0.0086) as an increased species in the MASLD group. In contrast, *Lachnospiraceae bacterium GAM79* (MASLD vs. Ctrl, 0.004 ± 0.009 vs. 0.018 ± 0.033, *p* = 0.014) was decreased in the MASLD group (Figure 2C).

In the LEfSe (LDA Effect Size) analysis, the MASLD and Ctrl groups had significantly different species in abundance. In the MASLD group, enriched species included *Bacillus* (genus), *Bacillales* (order), *Bacillaceae* (family), *Enterococcaceae* (genus), *Bifidobacterium longum* (species), *Enterococcus* (genus), and *Enterococcus faecium* (species). In the Ctrl group, enriched species included *Lachnospiraceae bacterium GAM79* (species), *Oscillospiraceae* (family), and *Ruminococcus* sp. *N15 MGS 57*(species) (Figure 2D). Similarly, in the phylogenetic tree, important microbial groups in the MASLD group were *Bacillaceae* (family), *Bacillales* (order), and *Enterococcaceae* (family). Important microbial groups in the Ctrl group were *Oscillospiraceae* (family) and *Clostridia* (order) (Figure 2E).

Considering *Parabacteroides merdae* inhabits the human gut, it specializes in branched-chain amino acid (BCAA) catabolism. We measured the concentration of glutamate in MASLD and Ctrl group. The result showed that the concentration of glutamate in MASLD are higher than that in Ctrl group (Figure 2F).

### 3.3 Functional prediction of gut microbiota in MASLD

**Figure 3:**
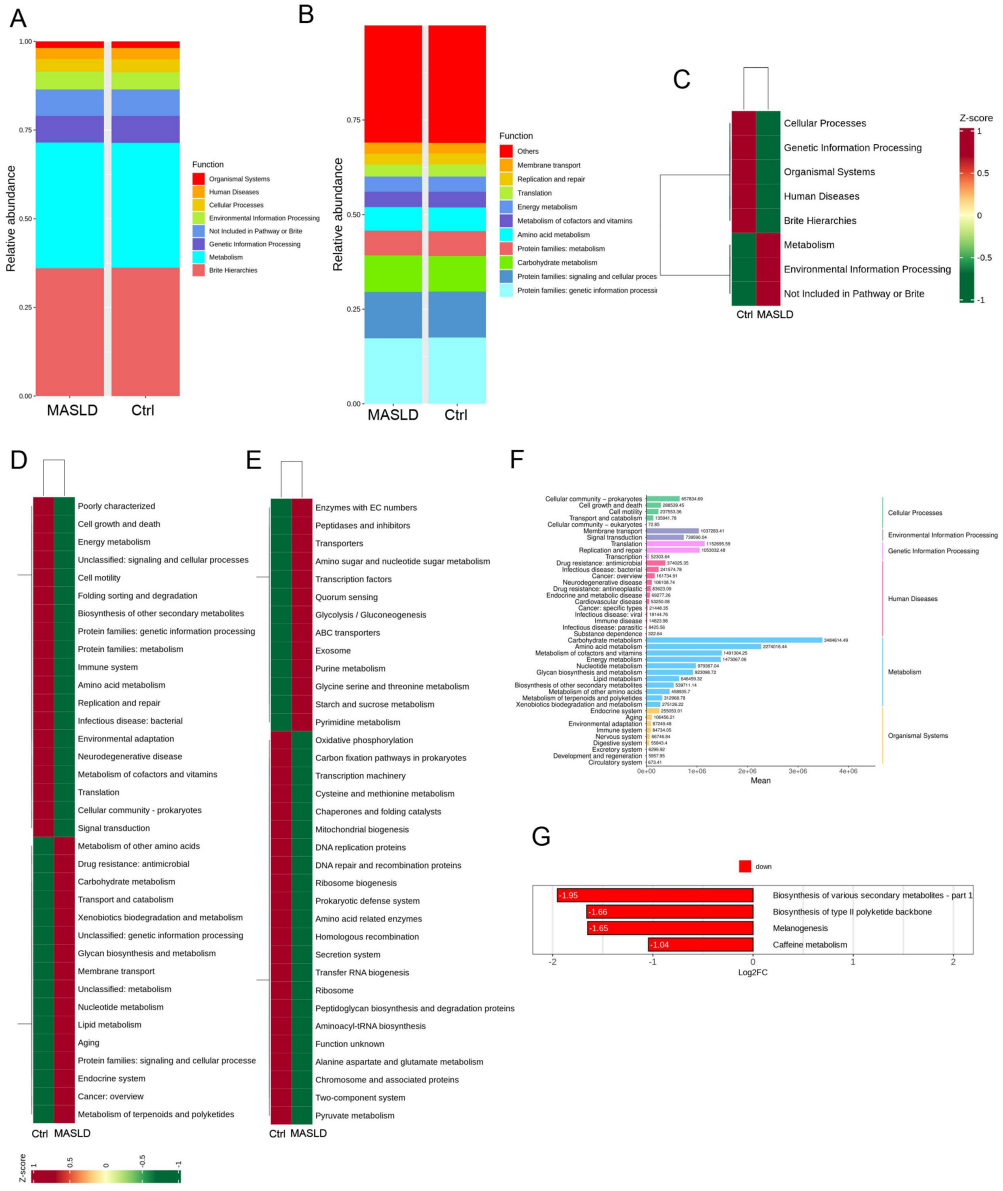
Functional prediction of gut microbiota in MASLD: **(A)** Bar chart of the top 10 most abundant function information distribution (level 1) between the MASLD and Ctrl groups; **(B)** Bar chart of the top 10 most abundant function information distribution (level 2) between the MASLD and Ctrl groups; **(C)** KEGG pathway enrichment analysis (level 1) between the MASLD and Ctrl groups; **(D)** KEGG pathway enrichment analysis (level 2) between the MASLD and Ctrl groups; **(E)** KEGG pathway enrichment analysis (level 3) between the MASLD and Ctrl groups; **(F)** Bar chart of enriched metabolic pathways in the MASLD group; **(G)** Bar chart of downregulated pathways in the Ctrl group.

The top 10 most abundant functions in the MASLD and Ctrl groups were analyzed. In level 1 analysis, both groups were enriched in Organismal Systems, Human Diseases, Cellular Processes, Environmental Information Processing, Not Included in Pathway or Brite, Genetic Information Processing, Metabolism, and Brite Hierarchies (Figure 3A). In level 2 analysis, both groups were enriched in Replication and repair, Membrane transport, Translation, Energy metabolism, Metabolism of cofactors and vitamins, Amino acid metabolism, Protein families: metabolism, Carbohydrate metabolism, Protein families: signaling and cellular processes, and Protein families: genetic information processing (Figure 3B).

KEGG clustering analysis was performed on the abundance functional pathways of the MASLD and Ctrl groups at three levels. At level 1, the MASLD group was enriched in Metabolism and Environmental Information Processing (Figure 3C). At level 2, the MASLD group was enriched in Metabolism of other amino acids, Drug resistance: antimicrobial, Carbohydrate metabolism, Transport and catabolism, Xenobiotics biodegradation and metabolism, Unclassified: genetic information processing, Glycan biosynthesis and metabolism, Membrane transport, Unclassified: metabolism, Nucleotide metabolism, Lipid metabolism, Aging, Protein families: signaling and cellular processes, Endocrine system, Cancer: overview, and Metabolism of terpenoids and polyketides (Figure 3D). At level 3, the MASLD group was enriched in Enzymes with EC numbers, Peptidases and inhibitors, Transporters, Amino sugar and nucleotide sugar metabolism, Transcription factors, Quorum sensing, Glycolysis/Gluconeogenesis, ABC transporters, Exosome, Purine metabolism, Glycine serine and threonine metabolism, Starch and sucrose metabolism, and Pyrimidine metabolism (Figure 3E).

In the KEGG pathway enrichment analysis, the enriched metabolic pathways included Carbohydrate metabolism, Energy metabolism, Lipid metabolism, Nucleotide metabolism, Amino acid metabolism, Metabolism of other amino acids, Glycan biosynthesis and metabolism, Metabolism of cofactors and vitamins, Metabolism of terpenoids and polyketides, Biosynthesis of other secondary metabolites, and Xenobiotics biodegradation and metabolism (Figure 3F). Compared with the MASLD group, the Ctrl group was downregulated in four pathways: Biosynthesis of type II polyketide backbone, Biosynthesis of various secondary metabolites, melanogenesis, and caffeine metabolism (Figure 3G).

### 3.4 Differential metabolite maps of untargeted metabolomics in MASLD

**Figure 4:**
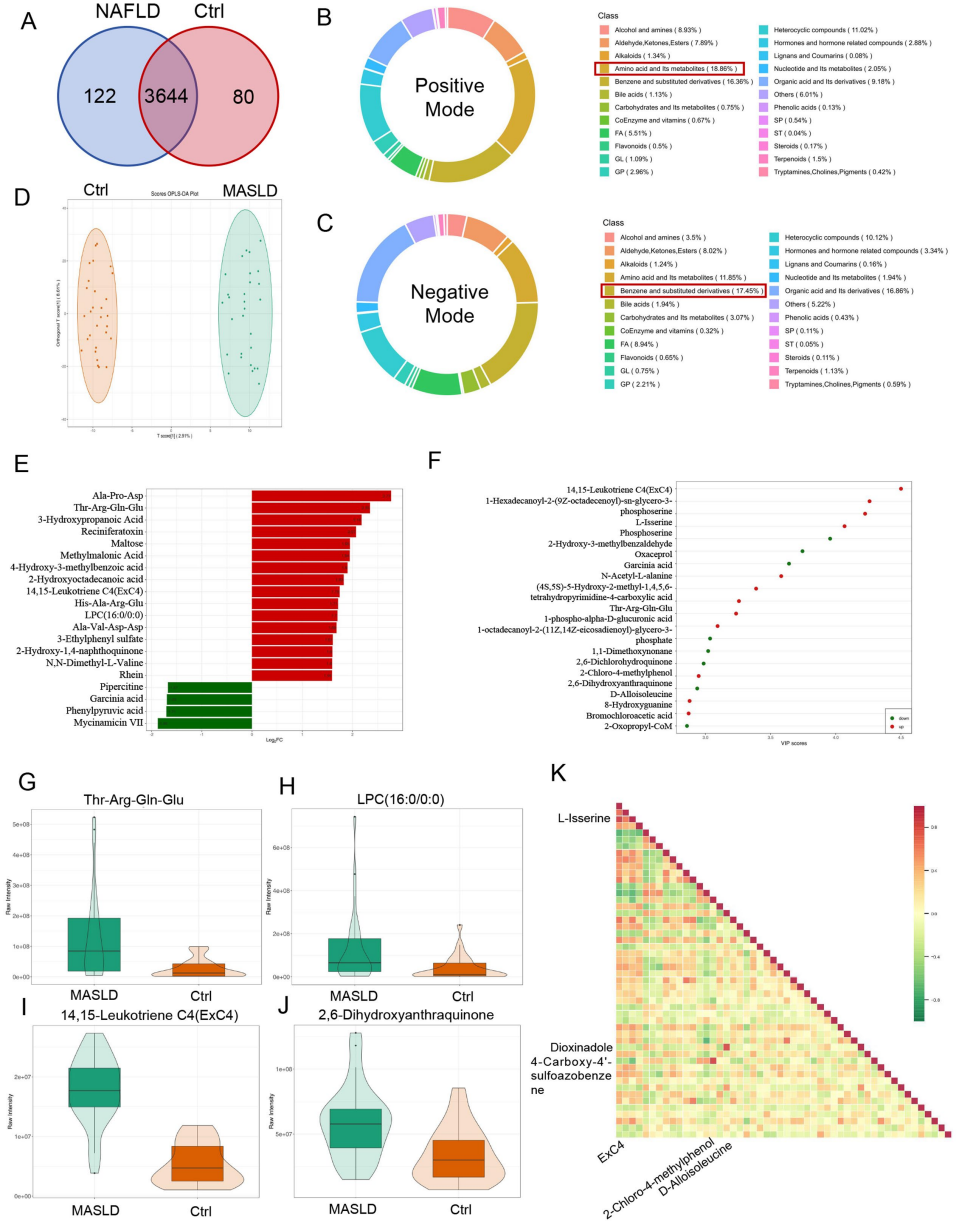
Differential metabolite maps of untargeted metabolomics in MASLD. **(A)** Venn diagram of differential metabolites between the MASLD and Ctrl groups; **(B)** Distribution proportion of metabolite types detected in positive mode; **(C)** Distribution proportion of metabolite types detected in negative mode; **(D)** OPLS-DA-based PCA analysis of the MASLD and Ctrl groups; **(E)** Bar chart of FCchanges in differential metabolites between the MASLD and Ctrl groups; **(F)** Dot plot of VIP score for differential metabolites between the MASLD and Ctrl groups; **(G–J)** Violin plots of differential metabolites including Thr-Arg-Gln-Glu, 14,15-Leukotriene C4 (ExC4), LPC (16:0/0:0), and 2,6-Dihydroxyanthraquinone; (K) Heatmap of correlation analysis of differential metabolites.

A total of 58 stool samples from the MASLD and Ctrl groups were collected for untargeted metabolomics analysis. Finally, 3,846 metabolites were detected, among which 3,209 metabolites were identified at the secondary level. Analysis of differential metabolites between the MASLD and Ctrl groups revealed 202 significantly different metabolites. Specifically, 80 metabolites were downregulated and 122 metabolites were upregulated in the MASLD group (Figure 4A). Under the positive mode, the most abundant metabolites were amino acid derivatives (18.86%), followed by benzene and substituted derivatives (16.36%) (Figure 4B). Under the negative mode, benzene and substituted derivatives were the most abundant (17.45%), followed by organic acid and its derivatives (16.36%) (Figure 4C). OPLS-DA was used to perform principal component analysis on the 58 samples, showing a significant separation between the metabolic profiles of the MASLD and Ctrl groups (Figure 4D).

Differential metabolites between the MASLD and Ctrl groups were screened using following criteria: VIP > 1 and *p*-value < 0.05. Metabolite tracing analysis was used to identify the metabolic functions of these metabolites based on their sources. The analysis of metabolite tracing mainly relied on the KEGG database, HMDB database, and CHEBI database.

Differential metabolite fold-change (FC) analysis was conducted between the MASLD and Ctrl groups. The top 16 upregulated metabolites mainly included amino acid and its metabolites (including Ala-Pro-Asp., Thr-Arg-Gln-Glu, His-Ala-Arg-Glu, Ala-Val-Asp-Asp., N,N-Dimethyl-L-Valine), organic acid and its derivatives (including 3-Hydroxypropanoic Acid, Methylmalonic Acid), benzene and substituted derivatives (including 4-Hydroxy-3-methylbenzoic acid, 2-Hydroxy-1,4-naphthoquinone, Rhein), GP [14,15-Leukotriene C4 (ExC4), LPC (16:0/0:0)], carbohydrates and its metabolites (including Maltose), Terpenoids (including Reciniferatoxin), FA (2-Hydroxyoctadecanoic acid), and others (3-Ethylphenyl sulfate). The top 4 downregulated metabolites included heterocyclic compounds (including Pipercitine, Mycinamicin VII), organic acid and its derivatives (including Garcinia acid, Phenylpyruvic acid) (Figure 4E). Differential metabolite analysis based on VIP score was performed between the MASLD and Ctrl groups. Upregulated metabolites included amino acid and its metabolites (including N-Acetyl-L-alanine, L-Isserine,(4S,5S)-5-Hydroxy-2-methyl-1,4,5,6-tetrahydropyrimidine-4-carboxylic acid, Thr-Arg-Gln-Glu, Phosphoserine), benzene and substituted derivatives (including 2,6-Dihydroxyanthraquinone), organic acid and its derivatives (including 1-phospho-alpha-D-glucuronic acid, Bromochloroacetic acid, 2-Oxopropyl-CoM), and GP (including 14,15-Leukotriene C4(ExC4), 1-Hexadecanoyl-2-(9Z-octadecenoyl)-sn-glycero-3-phosphoserine, 1-octadecanoyl-2-(11Z,14Z-eicosadienoyl)-glycerol-3-phosphate). Downregulated metabolites included organic acid and its derivatives (including Oxaceprol, Garcinia acid), Heterocyclic compounds (including 8-Hydroxyguanine), amino acid and its metabolites (including D-Alloisoleucine), others (1,1-Dimethoxynonane), and benzene and substituted derivatives (including 2-Hydroxy-3-methylbenzaldehyde,2,6-Dichlorohydroquinone, 2-Chloro-4-methylphenol) (Figure 4F).

The violin plots showed that Thr-Arg-Gln-Glu (Figure 4G), 14,15-Leukotriene C4(ExC4) (Figure 4H), LPC (16:0/0:0) (Figure 4I), and 2,6-Dihydroxyanthraquinone (Figure 4J) were all upregulated in the MASLD group. In the correlation analysis of differential metabolites, 14,15 - Leukotriene C4 (ExC4) was highly positively correlated with L-Isserine (r = 0.8688, *p* < 0.0001), 2-Chloro-4-methylphenol was highly positively correlated with 4-Carboxy-4′-sulfoazobenzene (*r* = 0.8515, *p* < 0.0001), and D-Alloisoleucine was highly positively correlated with Dioxindole (*r* = 0.8167, *p* < 0.0001) (Figure 4K).

### 3.5 Pathway enrichment analysis of differential metabolites in untargeted metabolomics

**Figure 5:**
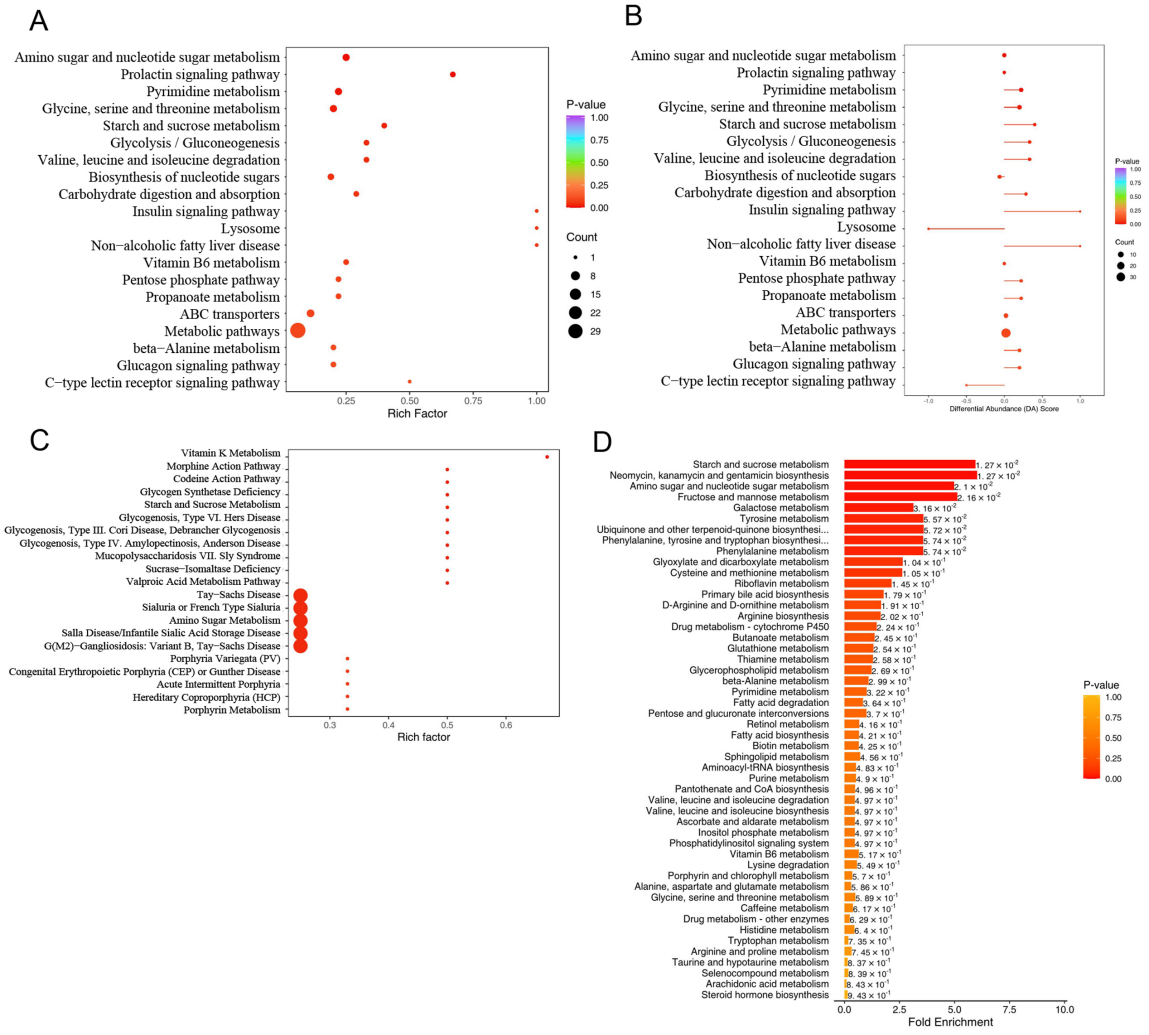
Pathway enrichment analysis of differential metabolites in untargeted metabolomics: **(A)** Dot plot of KEGG enrichment analysis for upregulated metabolites in MASLD; **(B)** Differential abundance scores of upregulated metabolites in MASLD; **(C)** Dot plot of HMDB functional annotation and enrichment analysis for upregulated metabolites in MASLD; **(D)** Bar chart of MSEA enrichment analysis for upregulated metabolites in MASLD.

KEGG pathway enrichment analysis was performed on the metabolites detected in the MASLD and Ctrl groups. The upregulated pathways in the MASLD group included Amino sugar and nucleotide sugar metabolism, Prolactin signaling pathway, Pyrimidine metabolism, Glycine, serine and threonine metabolism, Starch and sucrose metabolism, Glycolysis/Gluconeogenesis, Valine, leucine and isoleucine degradation, Biosynthesis of nucleotide sugars, Carbohydrate digestion and absorption, Insulin signaling pathway, Lysosome, Non-alcoholic fatty liver disease, Vitamin B6 metabolism, Pentose phosphate pathway, Propanoate metabolism, ABC transporters, Metabolic pathways, beta−Alanine metabolism, Glucagon signaling pathway, and C − type lectin receptor signaling pathway (Figure 5A). Subsequently, a pathway-based metabolic change analysis method was conducted according to the differential abundance score (DA Score). The DA Score can capture the overall changes of all metabolites in a pathway. The Insulin signaling pathway and Non − alcoholic fatty liver disease pathway were significantly upregulated (Figure 5B).

Then, functional annotation and enrichment analysis of differential metabolites were performed using the HMDB database. The upregulated metabolite pathways were enriched in Vitamin K Metabolism, Morphine Action Pathway, Codeine Action Pathway, Glycogen Synthetase Deficiency, Starch and Sucrose Metabolism, Glycogenosis, Type VI. Hers Disease, Glycogenosis, Type III. Cori Disease, Debrancher Glycogenosis, Glycogenosis, Type IV. Amylopectinosis, Anderson Disease, Mucopolysaccharidosis VII. Sly Syndrome, Sucrase-Isomaltase Deficiency, Valproic Acid Metabolism Pathway, Tay-Sachs Disease, Sialuria or French Type Sialuria, Amino Sugar Metabolism, Salla Disease/Infantile Sialic Acid Storage Disease, G(M2)-Gangliosidosis: Variant B, Tay-Sachs Disease, Porphyria Variegata (PV), Congenital Erythropoietic Porphyria (CEP) or Gunther Disease, Acute Intermittent Porphyria, Hereditary Coproporphyria (HCP), and Porphyrin Metabolism (Figure 5C).

MSEA enrichment analysis was used, and the enriched pathways included Starch and sucrose metabolism, Neomycin, kanamycin and gentamicin biosynthesis, Amino sugar and nucleotide sugar metabolism, Fructose and mannose metabolism, Galactose metabolism, Tyrosine metabolism, Ubiquinone and other terpenoid-quinone biosynthesis, Phenylalanine, tyrosine and tryptophan biosynthesis, Phenylalanine metabolism, and Glyoxylate and dicarboxylate metabolism. Multiple metabolic pathways, including Glycerophospholipid metabolism and Fatty acid degradation, were also included (Figure 5D).

### 3.6 Diagnostic efficacy of upregulated glycerophospholipid metabolites in MASLD

**Figure 6:**
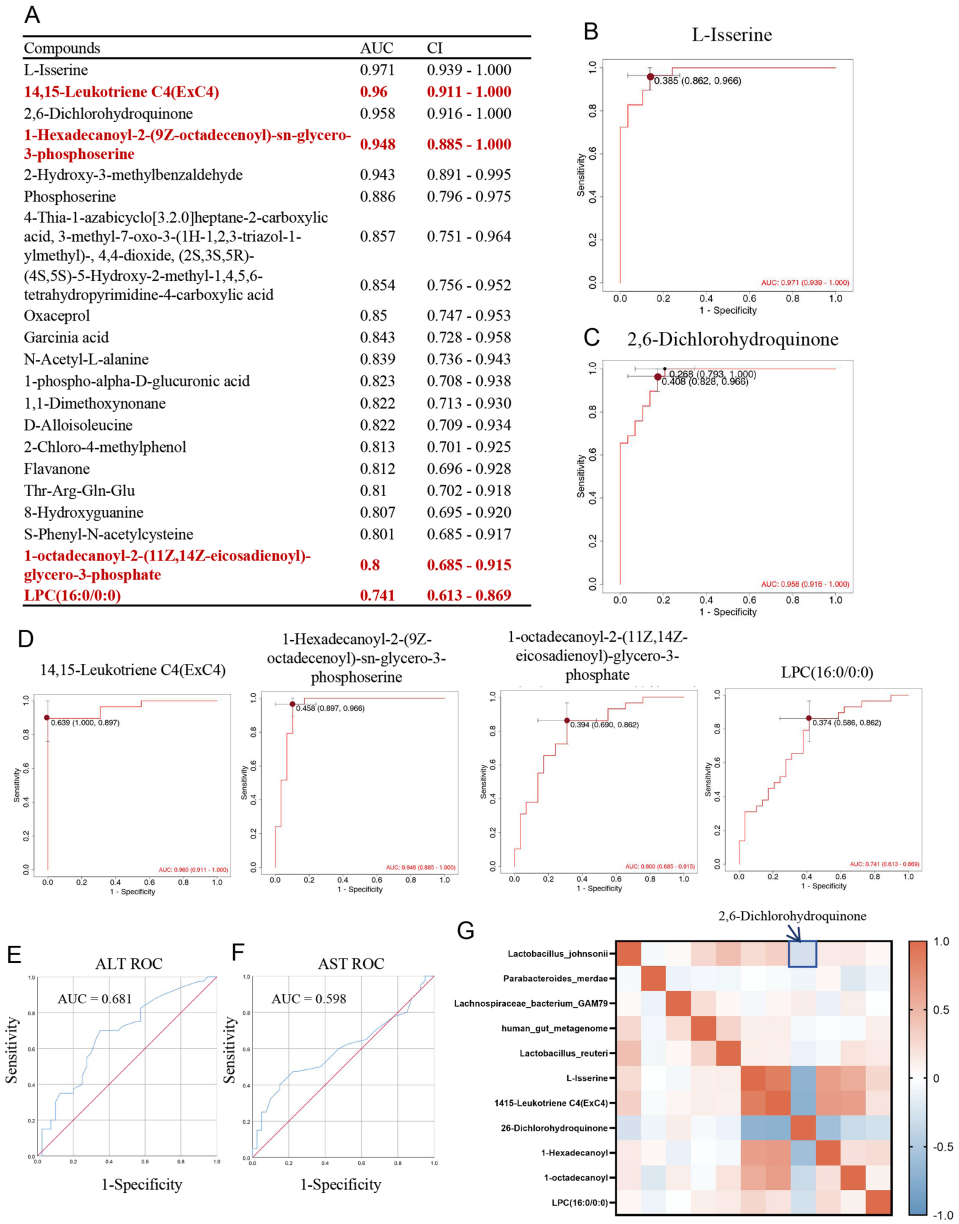
Diagnostic efficacy of upregulated glycerophospholipid metabolites in MASLD. **(A)** Table of AUC and CI for upregulated metabolites in MASLD; **(B)** ROC curve for L-Isserine; **(C)** ROC curve for 2,6-Dichlorohydroquinone; **(D)** ROC curves for glycerophospholipid metabolites 14,15-Leukotriene C4(ExC4), 1-Hexadecanoyl-2-(9Z-octadecenoyl)-sn-glycero-3-phosphoserine, 1-octadecanoyl-2-(11Z,14Z-eicosadienoyl)-glycerol-3-phosphate, and LPC (16:0/0:0); **(E)** ROC curve for ALT; **(F)** ROC curve for AST; **(G)** Correlation map between gut microbiota and metabolites.

In the MASLD group, Receiver Operating Characteristic (ROC)curve plottingand Area Under the Curve (AUC) were performed for upregulated metabolites. A total of 20 metabolites had AUC values greater than 0.8 (Figure 6A). Metabolites with AUC values greater than 0.95 included L-Isserine (AUC 0.971, CI 0.939–1.000, Youden index 0.385, specificity 0.966, sensitivity 0.862) (Figure 6B)and 2,6-Dichlorohydroquinone (AUC 0.958, CI 0.916–1.000, Youden index 0.408, specificity 0.966, sensitivity 0.828) (Figure 6C). Upregulated GPmetabolites all had AUC > 0.7, including 14,15-Leukotriene C4 (ExC4) (AUC 0.96, CI 0.911–1.000, Youden index 0.639, specificity 1.000, sensitivity 0.897), 1-Hexadecanoyl-2-(9Z-octadecenoyl)-sn-glycero-3- phosphoserine (AUC 0.948, CI 0.885–1.000, Youden index 0.458, specificity 0.897, sensitivity 0.966), 1-octadecanoyl-2-(11Z,14Z-eicosadienoyl)-glycerol-3-phosphate (AUC 0.80, CI 0.685–0.915, Youden index 0.394, specificity 0.690, sensitivity 0.862), and LPC (16:0/0:0) (AUC 0.74, CI 0.613–0.869, Youden index 0.374, specificity 0.586, sensitivity 0.862) (Figure 6D). And we compared the diagnostic performance of AST (AUC = 0.681) and ALT (AUC = 0.598) at Figures 6E,F. We added correlation analysis of gut microbiota and metabolites. The result showed that *Lactobacillus johnsonii* down-regulated in MASLD are negative correlation with 2,6-Dichlorohydroquinone (Figure 6G).

## 4 Discussion

MASLD still has a high incidence rate and remains a significant public health burden. It not only affects normal liver function but is also closely related to many serious health problems including NASH, liver fibrosis or cirrhosis, HCC, cardiovascular diseases. Therefore, we performed 16S rDNA sequencing on stool samples from 80 MASLD patients and healthy controls to analyze the composition of gut microbiota. We also conducted untargeted metabolomics on stool samples from 58 MASLD patients and healthy controls to characterize the gut microbiota metabolites in MASLD patients.

Firstly, we found that there is a characteristic of increased abundance of *Parabacteroides merdae* in MASLD. *Parabacteroides merdae*is one type of *Bacteroides.* Diet is an important factor affecting the abundance of *Bacteroides*. *Bacteroides* are commonly found in the gut of people living in Western countries (North America and Europe), because Western diets are usually high in fat and protein. MASLD patients also have a diet that is high in fat and protein, which is one of the main reasons for the increased abundance of *Parabacteroides merdae*. P*arabacteroides merdae* is a bacterium that commonly exists in the human gut. It mainly involves BCAA metabolism and can break down BCAAs such as leucine, isoleucine, and valine. And one intermediate is glutamate. BCAAs enter the cell via L-type amino acid transporters and reach the mitochondrion through SLC25A44. Inside the cell, branched-chain aminotransferase converts BCAAs into branched-chain *α*-keto acids (BCKAs) including α-ketoisocaproate (KIC), α-keto-*β*-methylvalerate (KMV), and α-ketoisovalerate (KIV). During this reaction, the amino group is transferred to α-ketoglutarate (α-KG), forming glutamate (Bezsudnova et al., 2017). Our result showed that the supernatant concentration of fecal suspension of glutamate in MASLD are higher than Ctrl. It showed gut microbiota in MASLD exhibits stronger BCAA metabolism than that in Ctrl. This helps reduce the levels of BCAAs in the blood, thereby decreasing the risk associated with insulin resistance and cardiovascular diseases. It also increases the production of short-chain fatty acids, regulates the immune system, and controls fat breakdown, blood glucose level, and energy utilization (Qiao et al., 2022). *Parabacteroides merdae* can protect cardiovascular tissue from atherosclerotic damage by enhancing the catabolism of BCAAs. It can also inhibit the mTORC1 signaling pathway in atherosclerotic plaques. Studies have shown that in models of obesity-and high-fat-diet-induced metabolic disorders, an increase in the abundance of *Parabacteroides merdae* is associated with improved insulin resistance and reduced fatty liver and other metabolic problems (Qiao et al., 2022). Therefore, the increased abundance of *Parabacteroides merdae* in the MASLD group may be a self-regulatory function of MASLD patients. It regulates the composition of the gut microbiota, increasing the abundance of beneficial bacteria (such as *Bifidobacterium*), thereby improving the balance of the gut microbiota. The upregulated bacteria in MASLD, including *Bacillus* and *Bifidobacterium longum*, may be involved in the self-regulatory process of *Parabacteroides merdae*. They can regulate the composition and structure of the gut microbiota, increase the abundance and composition of beneficial bacteria, and thus achieve self-metabolic regulation. B*acillus* has the function of regulating gut microbiota balance, improving digestive function, enhancing immunity, and alleviating inflammation and mucosal repair in the gut. Previous studies have shown that the DPP4-like enzyme of *Parabacteroides merdae* can simulate the proteolytic activity of human DPP4 on hormones such as peptide YY, neuropeptide Y, gastric inhibitory polypeptide (GIP), and glucagon-like peptide 1 (GLP-1). This is related to the upregulation of amino acids and their metabolites, including short peptides, in the untargeted metabolomics of MASLD (Flores Ramos et al., 2025). Thus, the high-fat, high-protein diet of MASLD patients is one of the reasons for the upregulation of *Parabacteroides merdae*. The upregulation of *Parabacteroides merdae* can regulate the catabolism of BCAAs in MASLD patients to some extent. It improves metabolic syndrome and increases the abundance of beneficial bacteria, including *Bifidobacterium*. Thisimproves the balance of the gut microbiota and is related to the upregulation of amino acids and their metabolites, including short peptides, in the untargeted metabolomics of MASLD.

In MASLD, there is also a characteristic downregulation of *Lachnospiraceae bacterium*. *Lachnospiraceae bacterium* is a widely-existing bacterium in the gut microbiota and has various important physiological functions and health benefits. Firstly, it has certain metabolic regulatory abilities. It can ferment dietary fiber to produce short-chain fatty acids such as acetate, propionate, and butyrate (Wolfe and Scharf, 2021). Moreover, *Lachnospiraceae bacterium* helps maintain the integrity of the intestinal barrier and regulate the structure of the mucus layer, thereby preventing the invasion of pathogens and harmful substances. In addition, the butyrate it produces can promote the growth and repair of colonic cells. By producing anti-inflammatory metabolites such as butyrate, *Lachnospiraceae bacterium* can regulate the host’s immune response and reduce intestinal inflammation. The abundance of *Lachnospiraceae bacterium* is closely related to the host’s metabolic health. Its presence in the gut is associated with reduced appetite and improved insulin sensitivity. In addition, it may indirectly affect the host’s metabolic state by regulating the composition of the gut microbiota (de Wouters d'Oplinter et al., 2022). It can also reduce liver damage by increasing the level of N-acetyl-glutamate and inhibiting ferroptosis. Moreover, its abundance is negatively correlated with the risk of developing metabolic diseases such as obesity and type 2 diabetes (Zhang et al., 2025). Therefore, the down-regulation of *Lachnospiraceae bacterium* in MASLD may lead to intestinal inflammatory responses in patients. Moreover, an increase in its abundance may lead to increased appetite in patients, thereby increasing insulin resistance and causing liver damage.

Therefore, we further explored the changes in MASLD metabolites through untargeted metabolomics. In the MASLD metabolomics analysis, we specifically identified upregulated glycerophospholipid metabolites in the MASLD group, including 14,15-Leukotriene C4 (ExC4), LPC (16:0/0:0), 1-Hexadecanoyl-2-(9Z-octadecenoyl)-sn-glycero-3-phosphoserine, and 1-octadecanoyl-2-(11Z,14Z-eicosadienoyl)-glycerol-3-phosphate. Multiple previous studies have shown that the increase in GPs in the gut is highly correlated with metabolic disorders and disease occurrence. The rise in glycerophospholipid metabolism may lead to lipid deposition in blood vessels, triggering cardiovascular diseases such as atherosclerosis (AS). In one study, the level of glycerophospholipid metabolism in the gut microbiota of the model group significantly increased, which was closely related to the occurrence of atherosclerosis (Song et al., 2025). Meanwhile, the upregulation of GPs in the gut may be associated with metabolic syndrome. For example, in the gut microbiota regulated by hypertriglyceridemia (HTG), glycerophospholipid metabolism is upregulated, which may affect the body’s metabolic status in a TLR4-dependent manner (Song et al., 2025). The upregulation of GPs may also be related to the regulation of the gut immune system. These metabolites can affect the immune response of the gut mucosa and regulate the level of inflammation (Qi et al., 2024). Especially in fatty liver disease, the upregulation of GPs may be associated with hepatic fat accumulation and inflammatory responses. Certain probiotics or plant extracts can improve glycerophospholipid metabolism by regulating the gut microbiota, thereby alleviating the symptoms of fatty liver disease (Yang et al., 2023). Therefore, regulating the glycerophospholipid metabolism of the gut microbiota can develop new therapeutic drugs for cardiovascular diseases, metabolic syndrome, and inflammatory diseases (Song et al., 2025). Considering this, we propose that the levels of glycerophospholipid metabolites in patient stool can be used for the diagnostic prediction of MASLD. The diagnostic AUC of GP metabolites is all greater than 0.7. Among them, 14,15-Leukotriene C4 (ExC4), 1-Hexadecanoyl −2-(9Z-octadecenoyl)-sn-glycero-3-phosphoserine, and 1-octadecanoyl-2-(11Z,14Z-eicosadienoyl) -glycerol-3-phosphate can even reach over 0.9. The increase in GP in the gut is highly correlated with metabolic disorders and disease occurrence, especially in MASLD, where the upregulation of GPs may be associated with hepatic fat accumulation and inflammatory responses. Therefore, the levels of glycerophospholipid metabolites in the stool of MASLD patients are good metabolic indicators for diagnosing MASLD and are potential targets for the diagnosis and treatment of MASLD.

And based on correlation analysis of gut microbiota and metabolites, *Lactobacillus johnsonii* down-regulated in MASLD are negative correlation with 2,6-Dichlorohydroquinone. It suggested *Lactobacillus johnsonii* exerts a potential detoxifying effect. It employs intracellular reductases to reduce 2,6-Dichlorohydroquinone and partially dechlorinate it, thereby attenuating its oxidative-stress toxicity (Kamaladevi et al., 2016; Han et al., 2024). So, Lack of *Lactobacillus johnsonii* in MASLD reduced detoxification capacity, and up-regulated 2,6-Dichlorohydroquinone by redox cycling that produces reactive oxygen species, caused lipid peroxidation and DNA damage.

**Figure 7:**
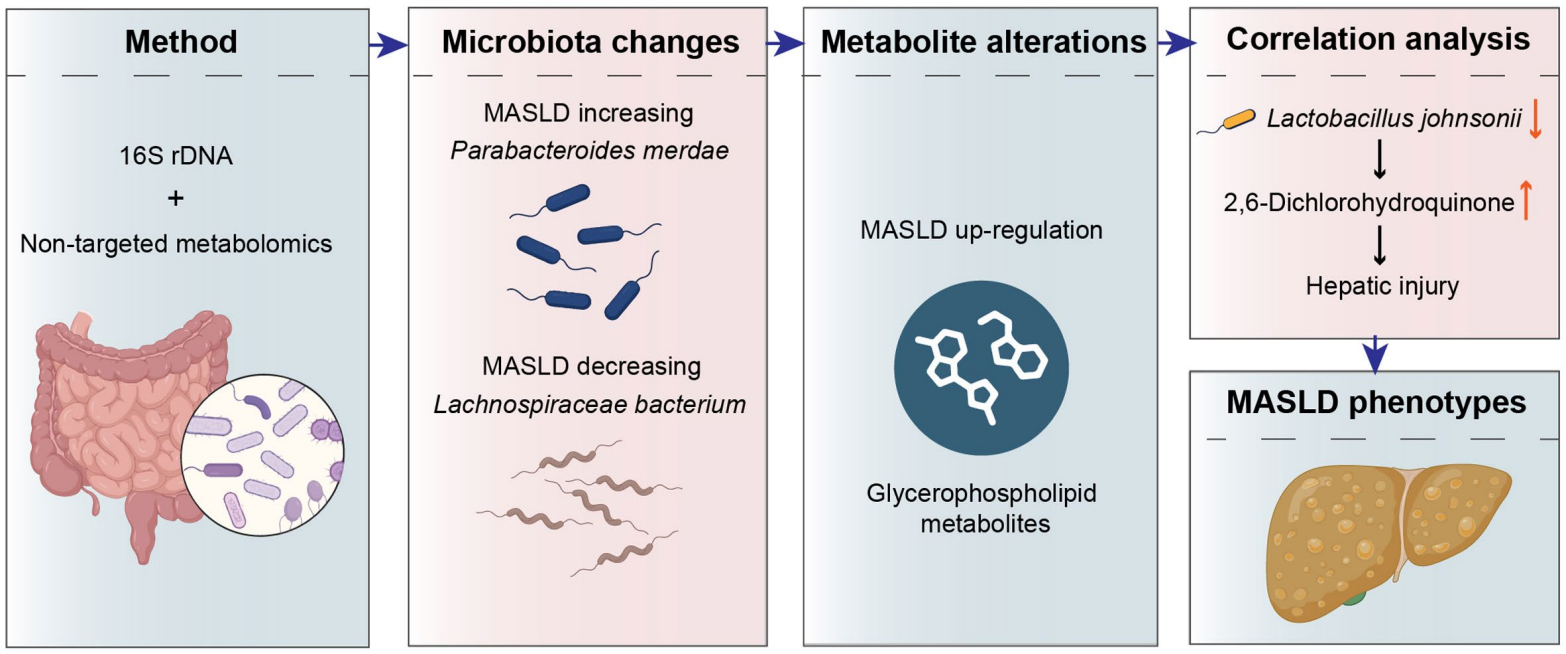
Mechanism schematic diagram of gut microbiota and metabolites in MASLD.

In conclusion, based on the high incidence rate of MASLD and its significant burden on public health, we aimed to explore the gut microbiota and metabolic maps of MASLD. We performed 16S rDNA sequencing on stool samples from 80 MASLD patients and healthy controls to analyze the composition of the gut microbiota. Additionally, we conducted untargeted metabolomics on stool samples from 58 MASLD patients and healthy controls to characterize the gut microbiota metabolites in MASLD patients. Ultimately, we identified relevant characteristics through the mapping of MASLD gut microbiota distribution. Firstly, the high-fat, high-protein diet of MASLD patients is one of the reasons for the increased abundance of *Parabacteroides merdae*. The upregulation of *Parabacteroides merdae* can enhance the catabolism of branched-chain amino acids in MASLD patients. It improves metabolic syndrome and increases the abundance of beneficial bacteria, including *Bifidobacterium*. Secondly, the down-regulation of *Lachnospiraceae bacterium* in MASLD may lead to intestinal inflammatory responses. An increase in its abundance may lead to increased appetite in patients, thereby increasing insulin resistance and causing liver damage. Then, the rise in GPs in the gut is highly correlated with metabolic disorders and disease occurrence. In particular, in MASLD, the up-regulation of GPs may be associated with hepatic fat accumulation and inflammatory responses. Therefore, the levels of glycerophospholipid metabolites in the stool of MASLD patients are good metabolic indicators for diagnosing fatty liver and are potential targets for the diagnosis and treatment of MASLD. And after conjoint analysis of gut microbiota and metabolites, we found that *Lactobacillus johnsonii* down-regulated in MASLD are negative correlation with 2,6-Dichlorohydroquinone. Lack of the *Lactobacillus johnsonii* drives 2,6-Dichlorohydroquinone accumulation, provoking toxic buildup and accelerating disease progression (Figure 7).

This study introduces three key innovations. First, we identified a MASLD-specific gut-microbiota signature featuring increased abundances of *Parabacteroides merdae* and decreased abundances of *Lachnospiraceae bacterium*. Second, GPs levels emerged as early, non-invasive biomarkers for MASLD, enabling at-home MASLD monitoring. Third, integrated microbiota-metabolite analysis revealed that lack of *Lactobacillus johnsonii* compromises detoxification. Hence, targeted supplementation of this bacterium can improve the gut microenvironment and modulate MASLD progression. Collectively, these findings define new microbiota traits, novel non-invasive biomarkers, and a beneficial bacterium for intervention for MASLD.

However, the current research still has some limitations. First, the up-regulation of glycerophospholipid metabolites was found in the gut. Whether there is also an increase in these metabolites in the blood of MASLD patients needs further validation. Second, whether glycerophospholipid metabolites can directly cause normal livers to develop into fatty livers needs to be verified *in vitro*, possibly using liver organoids. And, we found that the correlation between *Lactobacillus johnsonii* and the metabolite 2,6-dichlorohydroquinone suggests a potential detoxifying effect of the bacterium, it need a relevant functional validation in future work.

## Data Availability

The data presented in this study are publicly available. This data can be found at: https://ngdc.cncb.ac.cn/gsa, accession number CRA029645.
